# Long-Term Clinical Outcomes in Treatment-Naïve Patients With Orbital Adnexal Mucosa-Associated Lymphoid Tissue Lymphoma: A Single-Center Study

**DOI:** 10.3389/fonc.2022.769530

**Published:** 2022-05-06

**Authors:** Gi-June Min, Sung Eun Kim, Tong Yoon Kim, Young-Woo Jeon, Joo Hyun O, Byung-Ock Choi, Gyeongsin Park, Suk-Woo Yang, Seok-Goo Cho

**Affiliations:** ^1^ Department of Hematology, Catholic University Lymphoma Group, Seoul St. Mary’s Hospital, College of Medicine, The Catholic University of South Korea, Seoul, South Korea; ^2^ Department of Ophthalmology, Catholic University Lymphoma Group, Seoul St. Mary’s Hospital, College of Medicine, The Catholic University of South Korea, Seoul, South Korea; ^3^ Department of Hematology, Catholic University Lymphoma Group, Yeouido St. Mary’s Hospital, College of Medicine, The Catholic University of South Korea, Seoul, South Korea; ^4^ Department of Nuclear Medicine, Catholic University Lymphoma Group, Seoul St. Mary’s Hospital, College of Medicine, The Catholic University of South Korea, Seoul, South Korea; ^5^ Department of Radiation Oncology, Catholic University Lymphoma Group, Seoul St. Mary’s Hospital, College of Medicine, The Catholic University of South Korea, Seoul, South Korea; ^6^ Department of Hospital Pathology, Catholic University Lymphoma Group, Seoul St. Mary’s Hospital, College of Medicine, The Catholic University of South Korea, Seoul, South Korea

**Keywords:** orbital, lymphoma, primary therapy, long-term outcome, limited-stage

## Abstract

Ocular adnexal mucosa-associated lymphoid tissue (MALT) lymphoma (OAML) is the most common type of ocular lymphoma with a higher prevalence in Asia than in Western countries. OAML represents 1%–2% of all non-Hodgkin’s lymphoma, 5%–15% of extranodal lymphomas, and approximately 55% of orbital malignancies. “Watch and wait” after biopsy or surgical resection, radiation therapy, and systemic treatment, including antibiotics administration and chemotherapy with various combinations of regimens can be considered for OAML treatment. Radiotherapy is adapted for limited-stage disease with excellent clinical outcomes of 85–100% complete remission and relatively superior local control efficacy and treatment duration. In contrast, chemotherapy has rarely been tested as frontline therapy. Nonetheless, several studies have reported a favorable response and long duration of progression-free survival using chemotherapy adaptations. When the disease involves both eyes or spreads beyond the conjunctiva, the risk of recurrence increases and limited-stage OAML has a recurrence rate of approximately 25% following radiotherapy only. Therefore, although recent consensus in the literature is that patients with limited-stage OAML recommended treating with radiation, physicians may choose the treatment modality not only by its efficiency but also by its adverse events profile and patients’ well-being. Herein, we present a large single-center study on OAML that included 292 patients who were followed up for up to 237 months. We collected and analyzed real-world data focusing on treatment outcomes and the role of radiotherapy as frontline therapy, and aimed to compare outcomes and complication profiles of chemotherapy, especially in limited-stage OAML, to identify an optimal treatment strategy.

## Introduction

Ocular adnexal mucosa-associated lymphoid tissue (MALT) lymphoma (OAML) is the most common type of ocular lymphoma with a higher prevalence in Asia than in Western countries (1, 2). OAML represents 1%–2% of all non-Hodgkin’s lymphoma, 5%–15% of extranodal lymphomas, and approximately 55% of orbital malignancies (2–4). “Watch and wait” after biopsy or surgical resection, radiation therapy, and systemic treatment, including antibiotics administration and chemotherapy with various combinations of regimens can be considered for OAML treatment. However, there is no universally adapted therapy for OAML and other indolent marginal zone lymphomas except for such cases as gastric MALT lymphoma with *Helicobacter pylori* infection (5). Several retrospective studies reported that the “watch and wait” approach or antibiotic therapies have acceptable clinical outcomes but still have controversial issues with limited indications (6–10). Radiotherapy is adapted for limited-stage disease with excellent clinical outcomes of 85–100% complete remission and relatively superior local control efficacy and treatment duration (11). In contrast, chemotherapy has rarely been tested as a frontline therapy. Nonetheless, several studies have reported a favorable response and long duration of progression-free survival using chemotherapy adaptation (12–14). When the disease involves both eyes or spreads beyond the conjunctiva, the risk of recurrence increases, and limited-stage OAML has a recurrence rate of approximately 25% following radiotherapy only (3, 15–18). Therefore, although the recent consensus in the literature is that it is recommended that patients with limited-stage OAML be treated with radiation, physicians may choose the treatment modality not only by its efficiency but also by its adverse events profile and patients’ well-being. Herein, we present a large single-center study on OAML that included 292 patients who were followed up for up to 237 months. We collected and analyzed real-world data focusing on treatment outcomes and the role of radiotherapy as frontline therapy and aimed to compare outcomes and complication profiles of chemotherapy, especially in limited-stage OAML, to identify an optimal treatment strategy.

## Methods

### Patient Enrolment, Diagnosis, and Staging Workup

This single-center retrospective analysis included 292 patients diagnosed with OAML at the Catholic University Lymphoma Group between January 2004 and February 2020. OAML was diagnosed according to the morphological and immunophenotypic diagnostic criteria of lymphoma as defined by the World Health Organization (WHO) classification (19). Adequate Immunophenotyping of CD20 (+) with CD5 (-), CD10 (-), Cyclin-D1 (-) and demonstrating light chain restriction were performed with paraffin-embedded tissue specimen from the orbital adnexa lesion to diagnose MALT lymphoma and rule out other indolent lymphomas based on WHO classification. Expert pathologists confirmed the diagnosis of OAML made at the Catholic University Lymphoma Group, and experienced ophthalmologists at Catholic University Lymphoma Group performed tissue biopsies and further surgical treatment of OAML.

For staging work-up, we evaluated the routine imaging of the orbit area using magnetic resonance imaging and computed tomography of the neck, chest, abdomen, and pelvis, as well as 18-fluoro-2-deoxy-D-glucose (FDG) positron emission tomography (PET)-CT torso scan to detect distant lymph node or organ involvement. In our institution, bilateral iliac bone marrow crest biopsy is the routine and standard procedure for any lymphoma patients for staging work-up and initially conducted to identify bone marrow involvement in all patients suspected of having lymphoma regardless of its subtypes. All enrolled patients were stratified using the MALT-lymphoma International Prognostic Index (MALT-IPI) as reported (20), with the Ann-Arbor classification. Primary OAML was defined as a malignant neoplasm involving the conjunctiva, lacrimal gland, orbit, and eyelid. Therefore, bilateral ocular adnexal involvement was described as Ann-Arbor stage IE rather than IVE (21). We report according to the American Joint Committee on Cancer (AJCC) TNM staging system using orbital magnetic resonance imaging at the time of diagnosis, and as our group previously reported, we also identified patients who were more suitable for treatment strategies for localized OAML (15, 21). This AJCC TNM staging system is presented in **Table S1**.

### Treatment Strategy

The primary therapeutic modalities were determined using the TNM staging system combined with Ann-Arbor staging. Patients diagnosed with localized T1 or T2 stage with N0M0 or most Ann-Arbor stage IE generally underwent radiotherapy as first-line therapy. T3 to T4 with N0M0 patients were treated with either chemotherapy or radiotherapy according to the patients’ general status and the clinician’s decision. In the case of radiotherapy (n=179), the median treatment dose was 30 Gy (range, 20 to 40 Gy) with 1.8 to 2.0 Gy daily fraction size. Radiation dose was determined based on the location and extent of the lesion, and electron beams (5 to 10 MeV) were given to superficial lesions such as conjunctiva or eyelid. However, a 3D conformal plan and intensity-modulated radiotherapy were treated for deep-seated tumors. For lens protection, either a contact lens shield or hanging block was used in order not to reduce the target coverage. Target volume for any conjunctival or lacrimal glands mass lesions was defined as the entire conjunctiva, and for retrobulbar lesions, the whole orbital socket was treated. Patients with bilateral lesions were treated simultaneously.

Chemotherapy was administered to patients with an advanced Tx stage with N1 to N4 or M1, malignant cells involving bone marrow (n=44), or selected patients with bilateral involvement of the ocular adnexal area (n=53). The primary chemotherapy regimen was 8 cycles of R-CVP applied as the local standard, consisting of cyclophosphamide 750 mg/m^2^, vincristine 1.4 mg/m^2^, rituximab 375 mg/m^2^ on day 1, and prednisolone 60 mg/m^2^ on days 1–5 every 21 days. Several Ann-Arbor IE, T1 or T2 stage with N0M0 patients without any advanced nature also underwent chemotherapy because they refused treatment with radiotherapy for fear of ophthalmologic complications.

### Treatment Response and Adverse Events Assessment

All patients underwent a response evaluation every 3 months for 1 year, followed by every 6 months for 3 years, and then an annual check-up for local or systemic relapse by ophthalmologic and imaging studies. FDG PET-CT evaluation was always included in routine imaging workup if patients were initially diagnosed with extranodal involvements. However, if patients were diagnosed in localized OAML, only CT was performed to monitor systemic relapse. The following revised response criteria for malignant lymphoma were used for response assessment: complete remission was defined as the disappearance of all disease evidence, partial remission as at least 50% regression, but measurable remnant disease without a new lesion, progressive disease as any newly occurring lesion that had increased by more than 50% of the previously involved sites, and stable disease as the failure to attain complete remission, partial remission, or progressive disease (22). Adverse events related to chemotherapy or radiotherapy were assessed by an ophthalmologist, hematologist, and radiation oncologist according to the National Cancer Institute-Common Toxicity Criteria Adverse Events (version 5.0). Regular base questioning for subjective ophthalmologic symptoms, slit-lamp examination, visual field examination, Schirmer’s test, tear film break-up time, best-corrected visual acuity using the Snellen chart, and cataract evaluation using the Lens Opacity Classification System III was performed in radiotherapy-treated patients to assess ophthalmic complications.

### Ethical Approval

The study protocol was approved according to the guidelines of the institutional review board and ethics committee of the Catholic Medical Center, Republic of Korea. Consent for publication was not applicable for this study and was permitted by the institutional review board and ethics committee guidelines of the Catholic Medical Center (KC21RISI0358).

### Statistical Analysis

Overall survival was defined as the time from pathologic diagnosis to death or the last follow-up. Progression-free survival was calculated from the pathologic diagnosis until disease progression, transformation to aggressive lymphoma, relapse after complete remission, or death. Patients who remained disease-free at the time of the last follow-up were censored. Overall survival and progression-free survival were estimated using the Kaplan–Meier method, and the log-rank test was used to compare the differences between groups. Using cumulative incidence estimation, we calculated the cumulative incidence of relapse and non-relapsed mortality and compared the groups using Gray’s test. We treated death by any event without relapse and relapse incidence as competing risks for cumulative incidence of relapse and non-relapsed mortality calculations. Univariate analysis variables were selected based on prior literature on currently known factors or potential factors affecting survival outcomes according to the researcher’s prediction (13, 23). Multivariable analyses were performed using stepwise selection among candidate variables chosen from univariate analysis and excluding highly correlated variables. Demographic and clinical characteristics were analyzed using Student’s t-test and Chi-squared test. The R software version 3.4.1 (R Foundation for Statistical Computing, Vienna, Austria) was used for statistical analyses and a p-value <0.05 was considered statistically significant.

## Results

### Patients’ Characteristics and Clinical Manifestations

**Table 1** presents the demographic and clinical characteristics of the patients with OAML. The median age at diagnosis was 47 years (range, 18–84 years), and there were slightly more women (59.6%, n=174) than men (40.4%, n=118). OAML was diagnosed in the conjunctiva, orbital, eyelid, and lacrimal glands or ducts in 181 (61.9%), 58 (19.9%), 30 (10.3%), and 23 (7.9%) patients, respectively. At presentation, 247 (84.6%) patients had Ann-Arbor stage IE disease, including 159 (54.4%) patients with a TNM-AJCC stage T1N0M0 (n=104) or bT1N0M0 (n=55), representing OAML involving a single or both conjunctivas without orbital involvement. Moreover, 60 (20.5%), 24 (8.2%), and 4 (1.4%) patients presented with T2N0M0 involving the orbital area, T3N0M0 involving the preseptal eyelid, and T4N0M0 with lesions extending beyond the orbit to adjacent structures, respectively. Eighty-nine (30.5%) patients had bilateral involvement of ocular adnexal lesions, and 23 (7.9%) complained of B-symptoms. Advanced Ann-Arbor stage with extraocular involvement was present in 5 (1.7%) patients with stage II disease, 5 (1.7%) with stage III disease, and 35 (12.0%) with stage IV disease. The majority of patients (n=221, 75.7%) were classified as MALT-IPI low risk.

**Table 1:** Clinical characteristics of primary ocular adnexal MALT lymphoma (total n=292).

Characteristics	Values
**Age (years), median (range)**	47.0 (18 – 84)
Age ≥60 years	65 (22.3%)
Age ≥70 years	26 (8.9%)
**Sex**	
Male	118 (40.4%)
Female	174 (59.6%)
**Lactate dehydrogenase (**IU/L)**, median (range)**	347.0 (177.0 – 780.0)
Normal	272 (93.2%)
Elevated	20 (6.8%)
**B symptom**	
No	269 (92.1%)
Yes	23 (7.9%)
**ECOG performance status**	
0	260 (89.0%)
1	30 (10.3%)
2	2 (0.7%)
**Anatomical location (biopsy-proven)**	
Conjunctiva	181 (61.9%)^†^
Orbit	58 (19.9%)
Eyelid	30 (10.3%)
Lacrimal gland or duct	23 (7.9%)
**OD/OS/Both eye involvement**	109 (37.3%)/94 (32.2%)/89 (30.5%)
**Ann-Arbor stage/AJCC-TNM stage** ^‡^	
IE*	247 (84.6%)
* T1N0M0*	104 (35.6%)
* bT1N0M0* ^§^	55 (18.8%)
* T2N0M0*	60 (20.5%)
* T3N0M0*	24 (8.2%)
* T4N0M0*	4 (1.4%)
**II**	5 (1.7%)
* N1M0*	4 (80.0%)
* N2M0*	1 (20.0%)
**III**	5 (1.7%)
* N3M0*	3 (60.0%)
* N4M0*	2 (40.0%)
**IV**	35 (12.0%)
* M1a*	23 (65.7%)
* M1b*	1 (2.9%)
* M1c*	11 (31.4%)
**Bone marrow involvement**	12 (4.1%)
**Ki-67 ≥10%**	63 (21.6%)
**MALT-IPI score**	
Low risk (0)	221 (75.7%)
Intermediate risk (1)	58 (19.9%)
High risk (2-3)	13 (4.5%)

AJCC-TNM stage, American joint committee on cancer proposed the tumor, node, metastasis staging system; ECOG, eastern cooperative oncology group; IPI, international prognostic index; IU, international unit.

^†^The 167 patients were diagnosed with OMAL only in the conjunctiva, but the other 14 presented with conjunctiva and distant lymph node or extranodal organ involvement (1 with Ann-Arbor stage III and 13 with stage IV).

^‡^Detailed TNM clinical staging system description is presented in **Table SA**.

*Ann-Arbor IE represents a single isolated anatomical site of extranodal disease without nodal involvement or distant metastasis.

^§^Presenting bilateral conjunctival involvement without any other lesions outside the conjunctiva.

### Clinical Outcomes of Chemotherapy and Radiotherapy as First-Line Treatment

A total of 179 (59.3%) patients underwent radiotherapy as first-line therapy, including 178 patients with Ann-Arbor stage IE and one with Ann-Arbor stage II. By reclassifying the AJCC-TNM stage, 137 patients were either T1N0M0 or bT1N0M0, and the remaining 41 were beyond bT1N0M0 in the Ann-Arbor stage IE patient group. Overall, 158 (88.8%) patients who underwent radiotherapy with either ≥3,000 cGy (n=92, 51.7%) or <3,000 cGy (n=86, 48.3%) and achieved complete remission without relapse, which represented excellent outcomes of radiotherapy alone in limited-stage OAML. After radiotherapy, relapses were diagnosed from a median of 43 months (range, 12–91 months), and all 20 (11.2%) patients showed local relapse patterns without distant systemic relapse. Among the 20 relapsed patients, 13 presented with contralateral eye relapse. However, one patient with a third relapse found to be systemic, initially relapsed as a contralateral eye, eventually died due to refractoriness, and also possessed central nervous system lesions and bone marrow involvement of lymphoma with complex karyotype abnormalities.

Chemotherapy was used to treat 97 patients with OAML, of whom 53 (54.6%) were Ann-Arbor stage IE OAML. In the chemotherapy group of limited-stage, interim response evaluation was performed four cycles after R-CVP; 32 (60.4%) patients were in complete remission, and 21 (39.6%) patients were in either partial remission (n=20) or stable disease (n=1). However, 49 (92.5%) patients presented complete remission in response, and 4 (7.5%) remained in partial remission without stable disease or progressive disease, as the best response during follow-up. Ultimately, 44 (83.0%) patients achieved complete remission, and 9 (17.0%) relapsed after first-line chemotherapy. Among the 97 entirely chemotherapy-treated patients, 84 (86.6%) patients achieved complete remission, and 13 (13.4%) patients experienced a relapse. Relapse after frontline chemotherapy was diagnosed from a median of 41 months (range, 9–89 months) after treatment, and 9, 2, and 2 patients were initially Ann-Arbor stage IE, stage IIE, and stage IV, respectively. Six patients relapsed locally in the initially involved areas. Five patients relapsed locally advanced, beyond the initially involved field, and involving the eye socket. Two patients experienced recurrence at a distant location (spleen, kidney, cervical or mesenteric lymph nodes, and central nervous system). Locally relapsed patients (n=6) were treated with involved-field radiotherapy, and complete remission was achieved without treatment-related death or a third relapse. Relapsed as locally advanced (n=5) and systemic relapsed patients (n=2) were treated with salvage chemotherapy, two of whom were diagnosed with diffuse large B-cell lymphoma transformation. All patients with locally advanced relapse achieved complete remission after salvage chemotherapy. The responses to each first-line chemotherapy regimen and radiotherapy dose are summarized in **Table 2** and **Table S2**.

**Table 2:** The first-line treatment and clinical response stratified according to disease stage at diagnosis (total n=292).

Ann-Arbor stage	First-line treatment	Number of patients	Clinical response(Interim)	Clinical response(after treatment)	Systemic relapse	Local relapse	Others
**Stage IE (n=247)**	RT only	178	CR (176)/PR (1)/SD (1)	CR (176)/PR (2)	0	20 (contralateral 13)^†^	1^§^
	Chemotherapy	53	CR (32)/PR (20)/SD (1)	CR (49)/PR (4)	1*	8 (local 4; locally advanced 4)^‡^	0
	Surgical resection	16	CR (16)	CR (16)	0	0	0
**Stage II (n=5)**	RT only	1	CR (1)	CR (1)	0	0	0
	Chemotherapy	4	PR (4)	CR (3)/PR (1)	0	2 (local 1; locally advanced 1)	0
	Surgical resection	0	0		0	0	0
**Stage III (n=5)**	RT only	0	0		0	0	0
	Chemotherapy	5	CR (1)/PR (4)	CR (5)	0	0	0
	Surgical resection	0	0		0	0	0
**Stage IV (n=35)**	RT only	0	0		0	0	0
	Chemotherapy	35	CR (26)/PR (9)	CR (35)	1**	1 (local 1; locally advanced 0)	0
	Surgical resection	0	0		0	0	0
**Overall response**		292	CR (252)/PR (38)/SD (2)	CR (285)/PR (7) SD (0)/PD (0)	2 (0 died/2 alive)	31 (1 died/30 alive)	1 (1 died)

CR, complete remission; PR, partial remission; RT, radiotherapy.

*This patient relapsed as a high-grade transformation and finally achieved CR after six cycles of salvage chemotherapy with subsequent allogeneic hematopoietic transplantation and alive since.

**One systemic relapsed patient received Ibritumomab tiuxetan (ZEVALIN^®^) therapy after failure of several conventional salvage regimens and achieved CR and alive since.

^†^One patient experienced 3^rd^ relapse as CNS infiltration after successful local relapse treatment and eventually died.

^‡^Patients relapsed as locally advanced OAML compared to the initial stage who received salvage chemotherapy with rituximab and bendamustine.

^§^This patient was diagnosed with leiomyosarcoma and died without OAML relapse.

### Adverse Events of Each Primary Therapeutic Modality

Among 292 enrolled patients, 178 and 53 patients went through radiotherapy and chemotherapy as first-line treatment in limited-stage IE OAML, respectively. The other sixteen patients underwent surgical resection of OAML lesions only and achieved long-term complete remission. Permanent radiotherapy-related ophthalmic complications, including dry-eye syndrome (38.8%, median 4.2 months after beginning radiotherapy, range 0.8 to 12.3) and cataracts (26.4%, median 41.5 months, range 10.8 to 61.9), caused a persistent decline in quality of life (QoL). Twenty-three patients (12.9%) diagnosed with cataracts related to radiotherapy were less than 50 years old, and nineteen patients (10.7%) were treated with cataracts extraction surgery. Other significant ophthalmic complications included 25.3% of adnexal inflammation (median 2.0 months, range 0.5 to 9.1), 10.1% of radiation retinopathy (median 7.4 months, range 1.0 to 15.2), and 3.4% of nasolacrimal duct obstruction requiring surgical correction (median 3.8 months, range 1.5 to 9.8).

Conversely, the most significant chemotherapy-related complications were mainly temporal hematological adverse events. None of these adverse events lasted more than three months (median 26.5 days, range 4.5 to 86.7). Grade III-IV neutropenia (26.4%), anemia (5.7%), and thrombocytopenia (1.9%) were observed during chemotherapy as hematological adverse events. Among 14 patients who experienced Grade III-IV neutropenia during chemotherapy, only three patients required hospitalization to manage neutropenic fever. Grade III-IV hepatotoxicities (5.7%), acute renal injury (1.9%), and peripheral neuropathy (1.9%) were observed as non-hematological adverse events but were manageable and transient. Furthermore, both treatment modality groups did not show treatment-related mortality. **Tables 3** and **Table S3** list the adverse events after first-line treatment, either radiotherapy or chemotherapy for primary OAML in the limited-stage IE subgroup and the entire cohort.

**Table 3:** Adverse events by treatment modalities in Stage IE (n=231)^†^.

First-line radiotherapy (n=178)			First-line chemotherapy (n=53)					

Adverse events	Grade 1-2, N (%)	Grade 3-4, N (%)	Adverse events			Grade 1-2, N (%)		Grade 3-4, N (%)
Dry eyes	51 (28.7%)	18 (10.1%)	**Hematologic**		Neutropenia^§^	13 (24.5%)		14 (26.4%)
Cataract	22 (12.4%)	25 (14.0%)			Anemia	21 (39.6%)		3 (5.7%)
*Cataract diagnosis ≤50yrs*	14 (7.9%)	9 (5.0%)			Thrombocytopenia	7 (13.2%)		1 (1.9%)
*Cataract surgery*	0	19 (10.7%)	**Non-hematologic**		Hepatotoxicity	19 (35.9%)		3 (5.7%)
Adnexal inflammation^‡^	28 (15.7%)	17 (9.6%)			Acute renal injury	2 (3.8%)		1 (1.9%)
Retinopathy	12 (6.7%)	6 (3.4%)			Infection	7 (13.2%)		0 (0%)
Nasolacrimal duct obstruction	0	6 (3.4%)			Peripheral neuropathy*	16 (30.2%)		1 (1.9%)
**Therapy-related mortality**	0	0	**Therapy-related mortality**			0	0	

^†^Sixteen out of 247 limited-stage patients underwent surgical resection of OAML lesions only and achieved CR.

^‡^Adnexal inflammation includes keratitis, blepharitis, or conjunctivitis.

^§^Among 14 patients experienced Grade III-IV neutropenia during chemotherapy, 3 patients required hospitalization to manage neutropenic fever.

*There were no Grade IV peripheral neuropathies, which requires delayed chemotherapy, pain controlled by anesthetic intervention, and excluded vincristine in subsequent treatment regimen.

### Factors Affecting Clinical Outcomes

**Figure 1:**
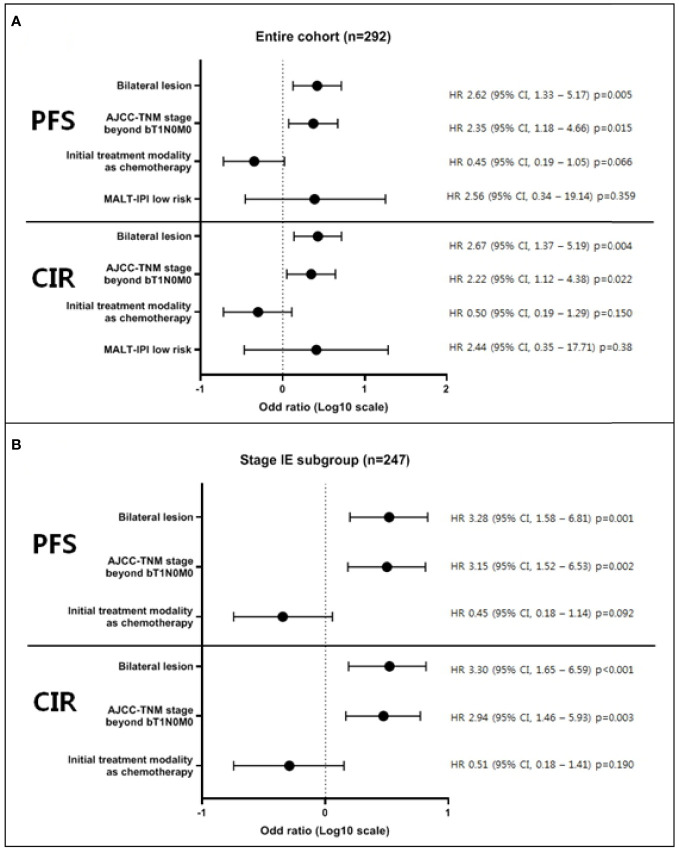
Multivariable analysis of factors affecting progression-free survival and cumulative incidence of relapse. Multivariable modes were derived using stepwise selection among candidate variables, based on evident factors from previous reports or researcher’s prediction, with the Wald test for overall p-value for factors with >2 levels and p-value <0.05 to warrant inclusion in the model. As a result, multivariable analysis showed the statistical significance of inferior progression-free survival and cumulative incidence of relapse in bilateral OAML involvement and initial TNM stage beyond bT1N0M0 in both the entire cohort **(A)** and stage IE subgroup **(B)**. All survival outcomes were defined as the time from pathologic diagnosis until each indicated time point (PFS; disease progression, transformation to aggressive lymphoma, relapse, or death, CIR; pathological diagnosis of relapse).

During a median follow-up of 68.1 months (range, 3.2–237.3 months), the overall survival and progression-free survival were 97.2% (95% confidence interval [CI], 89.3–99.3%), and 80.7% (95% CI, 73.1–86.4%), respectively. Cumulative incidence of relapse and non-relapsed mortality after completing the first-line treatment were 17.6% (95% CI, 12.2–23.9%) and 1.6% (95% CI, 0.1–7.7%), respectively. Associations between age, sex, location of lesions limited to conjunctivas or extended, bilaterality, stage, initial treatment modality, radiation dose, and progression free survival or cumulative incidence of relapse were evaluated. Univariate analyses of each survival outcome in the entire cohort and in the stage IE subgroup are presented in **Tables S4** and **S5**. Multivariable analysis showed the statistical significance of inferior progression-free survival in bilateral OAML involvement (hazard ratio [HR] = 2.62, *p* = 0.005 in the entire cohort and HR = 3.28, *p* = 0.001 in stage IE subgroup) and initial TNM stage beyond bT1N0M0 (HR = 2.35, *p* = 0.015 in the entire cohort and HR = 3.15, *p* = 0.002 in the stage IE subgroup) in both the entire cohort and stage IE subgroup. Multivariable analysis also showed similar results of significant inferior cumulative incidence of relapse in bilateral OAML involvement (HR = 2.67, *p* = 0.004 in the entire cohort and HR = 3.30, *p* < 0.001 in stage IE subgroup) and initial TNM stage beyond bT1N0M0 (HR = 2.22, *p* = 0.022 in the entire cohort and HR = 2.94, *p* = 0.003 in the stage IE subgroup). These results are presented in **Figure 1**.

## Discussion

We retrospectively analyzed 292 patients with OAML, with a median observation time of 68.1 months. A more extended follow-up period with 84 additional OAML patients was included compared with a previous report, and assessed patients treated with a homogeneous chemotherapy regimen of R-CVP or radiotherapy delivered a median dose of 30Gy (17 23). Although radiotherapy is the treatment of choice for limited-stage OAML, we focused on treatment outcomes in patients with limited-stage OAML using different frontline therapeutic modalities. OAML constitutes approximately 35%–80% of all ocular malignancies, and there is a higher proportion of primary OAML cases in Asian countries, such as Korea (2). Furthermore, in the Korean population, the initial presentation at a younger age with female predominance, mostly without distant organ involvement, is an important clinical characteristic of OAML (2, 15, 23).

The Ann-Arbor staging system often does not accurately reflect the stage of the extranodal organ involving lymphoma. To compensate for this limitation, the TNM staging system suggested by the AJCC includes additional staging categories based on greater detail, an accurate definition of local tumor extent, a description of multiple tumors, and a precise definition of regional nodal involvement concurrent with involvement of extranodal sites in the parotid or submandibular glands (21). Especially in limited-stage OAML, the accurate location of tumor involvement and its extent by TNM staging is related to a different prognosis (15). A previous study showed that bilaterality, non-conjunctival location, and nodal involvement were related to poor prognosis (16). In this study, 30.5% of patients had bilaterality, and 45.6% of them presented with non-conjunctival disease; accordingly, these patients had poor progression-free survival. A previous phase II study demonstrated that R-CVP was an efficient frontline regimen showing a durable response in limited-stage OAML with adverse factors, such as bilaterality or involvement beyond the conjunctiva (12). Furthermore, the clinical outcomes between the radiotherapy and chemotherapy group in this study showed no significant difference in the entire cohort and limited-stage OAML. Therefore, chemotherapy can be considered an alternative treatment option when physicians encountered cases of limited-stage but beyond bT1N0M0 or with bilateral involvement to prevent disease relapse or permanent radiotherapy-related complications.

Therapeutic strategies for OAML include surgical resection or biopsy with “watch and wait”, radiotherapy, and systemic treatment, including applying antibiotics and chemotherapy with various combinations of regimens. In our report, 16 patients diagnosed Ann-Arbor stage IE OAML with a localized unilateral conjunctival lesion of T1N0M0, and ophthalmologists decided to perform complete resection of the macroscopic lesion with careful observation. They remained in remission without relapse at a median of 32.4 months (range, 9.9-162.7 months) follow-up, suggesting that “watch and wait” might be a reasonable option after ruling out remnant lesions with potential ophthalmological damage. Previously, a few cases of the “watch and wait” approach have been reported with acceptable outcomes, but most of them were either from the refused initial treatment or asymptomatic OAML patients with uncertainty in selection criteria who might benefit without initial treatment (6, 7). Therefore, this issue warrants a prospective study. Several reports presented that antibiotics administration, such as doxycycline or clarithromycin, in OAML resulting a high response rate of up to 45-65% (8–10). However, the correlation between *Chlamydia psittaci* infection and OAML incidence is still controversial with geographic differences and not a universally adapted therapy as gastric MALT lymphoma (5, 16). Unfortunately, a *Chlamydia psittaci* infection study was not routinely performed in our institute; therefore, we did not analyze the clinical outcomes of antibiotics therapy.

Radiotherapy is currently the recommended first-line therapy in limited-stage OAML due to excellent treatment outcomes, low incidence of relapse, and the short treatment period of approximately three weeks without severe systemic adverse events. However, patients with OAML were relatively young (median age, 47 years), and radiotherapy as first-line therapy was more likely to lead to persistent complications, resulting in a lower QoL. Previous studies using radiotherapy demonstrated that the disease relapsed predominantly in an area that was not irradiated, mainly in the contralateral eye and rarely in distant extranodal organs (18, 24–26). In this study, 60.0% of relapsed OAML cases after RT were contralateral. In addition, there is controversy on the optimal radiation dose, and if the disease extends beyond the conjunctiva, the whole orbit should be covered during radiotherapy without lens shielding, thereby increasing the risk of cataracts considerably (27). Radiotherapy-related cataract may lead to early loss of near vision or other ophthalmic complications, including dry-eye syndrome and keratitis, resulting in a severe decline in QoL (28, 29). Furthermore, depending on the size and if involving sites in the orbit, the clinician could face patients complaining about radiotherapy induced ptosis (6.7%) or deformity of the orbital structure-related diplopia (5.1%). Several studies reported that low-dose radiotherapy with 4 Gy (2 Gy x 2) is effective and well-tolerated in patients affected with indolent non-Hodgkin lymphoma, including MALT lymphoma (30–33). OAML probably benefits the most from the 4 Gy protocol to avoid concomitant toxicities and achieve favorable outcomes. We did not have experience in low-dose radiotherapy of 4 Gy for OAML, but performing a prospective study to compare the safety and efficacy of conventional dosage versus low-dose radiotherapy in OAML will be a future task.

A wide range of chemotherapy regimens is available with or without anti-CD20 antibody rituximab for OAML or other indolent lymphomas (12, 14, 23, 34). In addition, rituximab monotherapy or investigational regimens, such as lenalidomide, were applied in the clinical trial setting (35–38). OAML patients also benefited from those chemotherapy, but a standard regimen is not currently established. Rituximab is an essential part of chemotherapy, and rituximab monotherapy or other combined regimens could be considered (such as CHOP consisting of cyclophosphamide, doxorubicin, vincristine, and prednisolone). However, the accredited standard chemotherapy regimen for indolent lymphoma was R-CVP, and national insurance covers only stage III or IV MALT lymphoma in Korea. Therefore, with our previous prospective study as evidence, we mainly utilize R-CVP regimen when considering systemic treatment in OAML patients (12). Although chemotherapy took a more extended treatment period than radiotherapy, requiring about six months to complete eight cycles of R-CVP, and should be carefully considered in patients with poor performance status or planning to become pregnant, chemotherapy demonstrated durable efficacy with more tolerable toxicity than radiotherapy and minimal delayed systemic or ophthalmologic adverse events. In addition, we routinely suggest sperm or ovum banking who decide to receive chemotherapy to release concerns about fertility issues in the younger patients. Hence, chemotherapy could be considered an alternative first-line therapy for relatively younger, limited Ann-Arbor stage, but beyond bT1N0M0 stage, patients with OAML, whereas radiotherapy could be reserved for older chemotherapy-ineligible or T1-bT1N0M0 stage patients.

There was a relatively small probability of events occurring with many censored patients because of the indolent nature of OAML with a long time-to-event, which may introduce bias in the study results. Therefore, the median follow-up duration of 68.1 months might not be sufficient to demonstrate the long-term course of this disease, especially for overall survival analysis. Although, given the fact that our study was retrospective and many previously known negative prognostic variables were not reproducible because of possible bias, relatively sufficient cohort size and the follow-up periods allowed us to analyze meaningful clinical parameters associated with distinguishing outcomes.

We demonstrated that Ann-Arbor limited-stage OAML patients should be re-staged based on AJCC-TNM staging and those with bilaterality or disease beyond the bT1N0M0 stage extending to orbital, eyelid, or eyelid adjacent structures beyond the orbit should be considered as a higher risk of relapse and poor progression-free survival. Since there were no clinical outcome differences between radiotherapy and chemotherapy in the limited-stage, these patients may carefully consider chemotherapy as an alternative to radiotherapy. Furthermore, younger patients who wish to avoid radiotherapy-related ophthalmic complications, often permanent, should also consider first-line chemotherapy. Radiotherapy can be reserved for older patients who may have difficulty bearing temporary but severe hematologic complications. In conclusion, chemotherapy may be regarded as a first-line treatment modality, except for patients with tumors limited to the uni- or bilateral conjunctiva or older OAML patients deemed unfit for chemotherapy.

## Data Availability Statement

The datasets presented in this article are not readily available because due to the nature of this research, participants of this study did not agree for their data to be shared publicly, so supporting data is not available. Requests to access the datasets should be directed to beichest@nate.com.

## Ethics Statement

The studies involving human participants were reviewed and approved by the institutional review board and ethics committee guidelines of the Catholic Medical Center (KC21RISI0358). Written informed consent for participation was not required for this study in accordance with the national legislation and the institutional requirements.

## Author Contributions

G-JM performed the research, collected and analyzed the data, and wrote the manuscript. SK, S-WY, B-OC provided patients and materials and reviewed the manuscript. Y-WJ, JO, and GP provided the materials and reviewed the manuscript. TK and S-GC reviewed the manuscript and analyzed the data. S-WY and S-GC designed and conducted the study, provided patients and materials, analyzed data, and wrote the manuscript. All authors read and approved the final manuscript.

## Conflict of Interest

The authors declare that the research was conducted in the absence of any commercial or financial relationships that could be construed as a potential conflict of interest.

## Publisher’s Note

All claims expressed in this article are solely those of the authors and do not necessarily represent those of their affiliated organizations, or those of the publisher, the editors and the reviewers. Any product that may be evaluated in this article, or claim that may be made by its manufacturer, is not guaranteed or endorsed by the publisher.

## References

[1] Yoon JS Ma KT Kim SJ Kook K Lee SY. Prognosis for Patients in a Korean Population With Ocular Adnexal Lymphoproliferative Lesions. Ophthalmic Plast Reconstr Surg (2007) 23:94–9. doi: 10.1097/IOP.0b013e318030b058 17413620

[2] Cho EY Han JJ Ree HJ Ko YH Kang YK Ahn HS. Clinicopathologic Analysis of Ocular Adnexal Lymphomas: Extranodal Marginal Zone B-Cell Lymphoma Constitutes the Vast Majority of Ocular Lymphomas Among Koreans and Affects Younger Patients. Am J Hematol (2003) 73:87–96. doi: 10.1002/ajh.10332 12749009

[3] Fung CY Tarbell NJ Lucarelli MJ Goldberg SI Linggood RM Harris NL. Ocular Adnexal Lymphoma: Clinical Behavior of Distinct World Health Organization Classification Subtypes. Int J Radiat Oncol Biol Phys (2003) 57:1382–91. doi: 10.1016/S0360-3016(03)00767-3 14630277

[4] Freeman C Berg JW Cutler SJ. Occurrence and Prognosis of Extranodal Lymphomas. Cancer (1972) 29:252–60. doi: 10.1002/1097-0142(197201)29:1<252::AID-CNCR2820290138>3.0.CO;2-# 5007387

[5] Zucca E Arcaini L Buske C Johnson PW Ponzoni M Raderer M. Marginal Zone Lymphomas: ESMO Clinical Practice Guidelines for Diagnosis, Treatment and Follow-Up. Ann Oncol (2020) 31:17–29. doi: 10.1016/j.annonc.2019.10.010 31912792

[6] Tanimoto K Kaneko A Suzuki S Sekiguchi N Maruyama D Kim SW. Long-Term Follow-Up Results of No Initial Therapy for Ocular Adnexal MALT Lymphoma. Ann Oncol (2006) 17:135–40. doi: 10.1093/annonc/mdj025 16236754

[7] Kiesewetter B Lukas J Kuchar A Mayerhoefer ME Streubel B Lagler H. Clinical Features, Treatment and Outcome of Mucosa-Associated Lymphoid Tissue (MALT) Lymphoma of the Ocular Adnexa: Single Center Experience of 60 Patients. PloS One (2014) 9:e104004. doi: 10.1371/journal.pone.0104004 25077481 PMC4117536

[8] Ferreri AJ Govi S Pasini E Mappa S Bertoni F Zaja F. Chlamydophila Psittaci Eradication With Doxycycline as First-Line Targeted Therapy for Ocular Adnexae Lymphoma: Final Results of an International Phase II Trial. J Clin Oncol (2012) 30:2988–94. doi: 10.1200/JCO.2011.41.4466 22802315

[9] Ferreri AJM Dognini GP Ponzoni M Pecciarini L Cangi MG Santambrogio G. Chlamydia Psittaci-Eradicating Antibiotic Therapy in Patients With Advanced-Stage Ocular Adnexal MALT Lymphoma. Ann Oncol (2008) 19:194–5. doi: 10.1093/annonc/mdm561 18073219

[10] Ferreri AJM Ponzoni M Guidoboni M Resti AG Politi LS Cortelazzo S. Bacteria-Eradicating Therapy With Doxycycline in Ocular Adnexal MALT Lymphoma: A Multicenter Prospective Trial. JNCI (2006) 98:1375–82. doi: 10.1093/jnci/djj373 17018784

[11] Lee JL Kim MK Lee KH Hyun MS Chung HS Kim DS. Extranodal Marginal Zone B-Cell Lymphomas of Mucosa-Associated Lymphoid Tissue–Type of the Orbit and Ocular Adnexa. Ann Hematol (2005) 84:13–8. doi: 10.1007/s00277-004-0914-3 15309523

[12] Kim SY Yang SW Lee WS Yang JW Oh SY Ahn HB. Frontline Treatment With Chemoimmunotherapy for Limited-Stage Ocular Adnexal MALT Lymphoma With Adverse Factors: A Phase II Study. Oncotarget (2017) 8:68583–90. doi: 10.18632/oncotarget.19788 PMC5620279 28978139

[13] Desai A Joag MG Lekakis L Chapman JR Vega F Tibshirani R. Long-Term Course of Patients With Primary Ocular Adnexal MALT Lymphoma: A Large Single-Institution Cohort Study. Blood (2017) 129:324–32. doi: 10.1182/blood-2016-05-714584 27789481

[14] Song EK Kim SY Kim TM Lee KW Yun T Na II. Efficacy of Chemotherapy as a First-Line Treatment in Ocular Adnexal Extranodal Marginal Zone B-Cell Lymphoma. Ann Oncol (2008) 19:242–6. doi: 10.1093/annonc/mdm457 17947227

[15] Lee SE Paik JS Cho WK Choi BO Lee SN Jung SE. Feasibility of the TNM-Based Staging System of Ocular Adnexal Extranodal Marginal Zone Lymphoma of Mucosa-Associated Lymphoid Tissue (MALT Lymphoma). Am J Hematol (2011) 86:262–6. doi: 10.1002/ajh.21963 21328439

[16] Stefanovic A Lossos IS. Extranodal Marginal Zone Lymphoma of the Ocular Adnexa. Blood (2009) 114:501–10. doi: 10.1182/blood-2008-12-195453 PMC2713468 19372259

[17] Nam H Ahn YC Kim YD Ko Y Kim WS. Prognostic Significance of Anatomic Subsites: Results of Radiation Therapy for 66 Patients With Localized Orbital Marginal Zone B Cell Lymphoma. Radiother Oncol (2009) 90:236–41. doi: 10.1016/j.radonc.2008.09.011 18950885

[18] Uno T Isobe K Shikama N Nishikawa A Oguchi M Ueno N. Radiotherapy for Extranodal, Marginal Zone, B-Cell Lymphoma of Mucosa-Associated Lymphoid Tissue Originating in the Ocular Adnexa. Cancer (2003) 98:865–71. doi: 10.1002/cncr.11539 12910532

[19] International Agency for Research on Cancer, World Health Organization. WHO Classification of Tumours of Haematopoietic and Lymphoid Tissues. In: Swerdlow SH Campo E Harris NL Jaffe ES Pileri SA Stein H Thiele J, editors. WHO Classification of Tumors, Revised Fourth Ed, vol. Volume 2. Lyon, France: International Agency for Research on Cancer (2017). p. 259 p.

[20] Thieblemont C Cascione L Conconi A Kiesewetter B Raderer M Gaidano G. A MALT Lymphoma Prognostic Index. Blood (2017) 130:1409–17. doi: 10.1182/blood-2017-03-771915 28720586

[21] Finger PT 7th Edition, AJCC-UICC Ophthalmic Oncology Task Force. The 7th Edition AJCC Staging System for Eye Cancer: An International Language for Ophthalmic Oncology. Arch Pathol Lab Med (2009) 133:1197–8. doi: 10.5858/133.8.1197 19653708

[22] Cheson BD Pfistner B Juweid ME Gascoyne RD Specht L Horning SJ. International Harmonization Project on Lymphoma Revised Response Criteria for Malignant Lymphoma. J Clin Oncol (2007) 25:579–86. doi: 10.1200/JCO.2006.09.2403 17242396

[23] Jeon YW Yang HJ Choi BO Jung SE Park KS JH O. Comparison of Selection and Long-Term Clinical Outcomes Between Chemotherapy and Radiotherapy as Primary Therapeutic Modality for Ocular Adnexal MALT Lymphoma. EClinicalMedicine (2018) 4-5:32–42. doi: 10.1016/j.eclinm.2018.10.001 31193655 PMC6537565

[24] Raderer M Streubel B Woehrer S Puespoek A Jaeger U Formanek M. High Relapse Rate in Patients With MALT Lymphoma Warrants Lifelong Follow-Up. Clin Cancer Res (2005) 11:3349–52. doi: 10.1158/1078-0432.CCR-04-2282 15867234

[25] Martinet S Ozsahin M Belkacémi Y Landmann C Poortmans P Oehlere C. Outcome and Prognostic Factors in Orbital Lymphoma: A Rare Cancer Network Study on 90 Consecutive Patients Treated With Radiotherapy. Int J Radiat Oncol Biol Phys (2003) 55:892–8. doi: 10.1016/S0360-3016(02)04159-7 12605966

[26] Lee SW Suh CO Kim GE Yang WI Lee SY Hahn JS. Role of Radiotherapy for Primary Orbital Lymphoma. Am J Clin Oncol (2002) 25:261–5. doi: 10.1097/00000421-200206000-00011 12040284

[27] Woolf DK Kuhan H Shoffren O Akinnawo EM Sivagurunathan B Boyce H. Outcomes of Primary Lymphoma of the Ocular Adnexa (Orbital Lymphoma) Treated With Radiotherapy. Clin Oncol (2015) 27:153–9. doi: 10.1016/j.clon.2014.10.002 25455843

[28] Cho WK Lee SE Paik JS Cho SG Yang SW. Risk Potentiality of Frontline Radiotherapy Associated Cataract in Primary Ocular Adnexal Mucosa-Associated Lymphoid Tissue Lymphoma. Korean J Ophthalmol (2013) 27:243–8. doi: 10.3341/kjo.2013.27.4.243 PMC3730065 23908569

[29] Tanimoto K Kaneko A Suzuki S Sekiguchi N Watanabe T Kobayashi Y. Primary Ocular Adnexal MALT Lymphoma: A Long-Term Follow-Up Study of 114 Patients. Japanese J Clin Oncol (2007) 37:337–44. doi: 10.1093/jjco/hym031 17562719

[30] Ganem G Lambin P Socié G Girinsky T Bosq J Pico JL. Potential Role for Low Dose Limited-Field Radiation Therapy (2 X 2 Grays) in Advanced Low-Grade non-Hodgkin's Lymphomas. Hematol Oncol (1994) 12:1–8. doi: 10.1002/hon.2900120102 8194839

[31] Hoskin PJ Kirkwood AA Popova B Smith P Robinson M Gallop-Evans E. 4 Gy Versus 24 Gy Radiotherapy for Patients With Indolent Lymphoma (Fort): A Randomised Phase 3 non-Inferiority Trial. Lancet Oncol (2014) 15:457–63. doi: 10.1016/S1470-2045(14)70036-1 24572077

[32] Hoskin P Popova B Schofield O Brammer C Robinson M Brunt AM. 4 Gy Versus 24 Gy Radiotherapy for Follicular and Marginal Zone Lymphoma (Fort): Long-Term Follow-Up of a Multicentre, Randomised, Phase 3, non-Inferiority Trial. Lancet Oncol (2021) 22:332–40. doi: 10.1016/S1470-2045(20)30686-0 33539729

[33] Cerrato M Orlandi E Vella A Bartoncini S Iorio GC Bongiovanni D. Efficacy of Low-Dose Radiotherapy (2 Gy × 2) in the Treatment of Marginal Zone and Mucosa-Associated Lymphoid Tissue Lymphomas. Br J Radiol (2021) 94:20210012. doi: 10.1259/bjr.20210012 34111959 PMC8248200

[34] Paik JS Cho WK Lee SE Choi BO Jung SE Park GS. Ophthalmologic Outcomes After Chemotherapy and/or Radiotherapy in non-Conjunctival Ocular Adnexal MALT Lymphoma. Ann Hematol (2012) 91:1393–401. doi: 10.1007/s00277-012-1469-3 22543827

[35] Rummel MJ Niederle N Maschmeyer G Banat GA von Grünhagen U Losem C. Bendamustine Plus Rituximab Versus CHOP Plus Rituximab as First-Line Treatment for Patients With Indolent and Mantle-Cell Lymphomas: An Open-Label, Multicentre, Randomised, Phase 3 non-Inferiority Trial. Lancet (2013) 381:1203–10. doi: 10.1016/S0140-6736(12)61763-2 23433739

[36] Barbara K Marlene T Werner D Leonhard M Julius L Christoph CZ. A Phase II Study of Lenalidomide in Patients With Extranodal Marginal Zone B-Cell Lymphoma of the Mucosa Associated Lymphoid Tissue (MALT Lymphoma). Haematologica (2013) 98:353–6. doi: 10.3324/haematol.2012.065995 PMC3659944 22899582

[37] Conconi A Martinelli G Thiéblemont C Ferreri AJ Devizzi L Peccatori F. Clinical Activity of Rituximab in Extranodal Marginal Zone B-Cell Lymphoma of MALT Type. Blood (2003) 102:2741–5. doi: 10.1182/blood-2002-11-3496 12842999

[38] Troch M Kiesewetter B Willenbacher W Willenbacher E Zebisch A Linkesch W. Rituximab Plus Subcutaneous Cladribine in Patients With Extranodal Marginal Zone B-Cell Lymphoma of Mucosa-Associated Lymphoid Tissue: A Phase II Study by the Arbeitsgemeinschaft Medikamentose Tumortherapie. Haematologica (2013) 98:264–8. doi: 10.3324/haematol.2012.072587 PMC3561434 22983582

