# Supplementary material for: Long-Term Clinical Outcomes in Treatment-Naïve Patients With Orbital Adnexal Mucosa-Associated Lymphoid Tissue Lymphoma: A Single-Center Study
Source: Front Oncol. 2022 May 6;12:769530. doi: 10.3389/fonc.2022.769530 (PMC9120944; doi:10.3389/fonc.2022.769530)
Supplement: Supplementary file 1 [file Table_1.docx]

Supplementary Material

# Supplementary Table

**Table S1. TNM Clinical Staging for ocular adnexal MALT lymphoma (OAMLs)***

| **TNM Stage** | **Description** | |
| --- | --- | --- |
| **Primary tumor (T)** | | |
| **TX** | | Lymphoma extent not specified |
| **T0** | | No evidence of lymphoma |
| **T1** | | Lymphoma involving the conjunctiva alone without orbital involvement |
| **T1a** | | Bulbar conjunctiva only |
| **T1b** | | Palpebral conjunctiva ± fornix ± caruncle |
| **T1c** | | Bulbar and nonbulbar conjunctival involvement |
| **T2** | | Lymphoma with orbital involvement ± any conjunctival involvement |
| **T2a** | | Anterior orbital involvement†, but no lacrimal gland involvement (± conjunctival disease) |
| **T2b** | | Anterior orbital involvement with lacrimal gland involvement (± conjunctival disease) |
| **T2c** | | Posterior orbital involvement (± conjunctival involvement ± any extraocular muscle involvement) |
| **T2d** | | Nasolacrimal drainage system involvement (± conjunctival involvement but not including nasopharynx) |
| **T3** | | Lymphoma with preseptal eyelid involvement‡ ± orbital involvement ± any conjunctival involvement |
| **T4** | | Orbital adnexal lymphoma extending beyond orbit to adjacent structures, such as bone and brain |
| **T4a** | | Involvement of nasopharynx |
| **T4b** | | Osseous involvement (including periosteum) |
| **T4c** | | Involvement of maxillofacial, ethmoidal ± frontal sinuses |
| **T4d** | | Intracranial spread |
| **Lymph node involvement (N)** | | |
| **NX** | | Involvement of lymph nodes not assessed |
| **N0** | | No evidence of lymph node involvement |
| **N1** | | Involvement of ipsilateral regional lymph nodes§ |
| **N2** | | Involvement of contralateral or bilateral regional lymph nodes§ |
| **N3** | | Involvement of peripheral lymph nodes not draining ocular adnexal region** |
| **N4** | | Involvement of central lymph nodes** |

| **Distant metastasis (M)** | |
| --- | --- |
| **MX** | Dissemination of lymphoma not assessed |
| **M0** | No evidence of involvement of other extranodal sites |
| **M1** | Lymphomatous involvement in other organs recorded either at first diagnosis or subsequently |
| **M1a** | Noncontiguous involvement of tissues or organs external to the ocular adnexa (eg, parotid glands, submandibular gland, lung, liver, spleen, kidney, breast) |
| **M1b** | Lymphomatous involvement of the bone marrow |
| **M1c** | Both M1a and M1b involvement |

* Used with the permission of the American Joint Committee on Cancer (AJCC), Chicago, Illinois. The original source for this material is the AJCC Cancer Staging Manual, Seventh Edition (2009) published by Springer Science and Business Media LLC, [www.springerlink.com](http://www.springerlink.com).

Ref.) S.E. Coupland, V.A. White, J. Rootman, B. Damato, P.T. Finger, A TNM-Based Clinical Staging System of Ocular Adnexal Lymphomas, Archives of pathology & laboratory medicine 133(8) (2009) 1262-1267.

† The anterior orbit is defined as the area between the orbital septum and the equator of the globe. The posterior orbit is defined as the area posterior to the equator of the globe, extending to the orbital apex.

‡ Eyelid involvement is said to exist when the OAL infiltrates preseptal tissues (ie, tissues anterior to the orbital septum).

§ Regional lymph nodes, which include the preauricular (parotid), submandibular, and cervical lymph nodes.

** Distant nodes include ‘‘central’’ nodes, located in the trunk (eg, mediastinal and para-aortic nodes) and ‘‘peripheral’’ nodes at other distant sites not draining the ocular adnexa (eg, popliteal lymph nodes). The different substages of N indicate whether the lymph node involvement is local (ipsilateral, contralateral, bilateral), central, or peripheral.
