# Supplementary material for: Long-Term Clinical Outcomes in Treatment-Naïve Patients With Orbital Adnexal Mucosa-Associated Lymphoid Tissue Lymphoma: A Single-Center Study
Source: Front Oncol. 2022 May 6;12:769530. doi: 10.3389/fonc.2022.769530 (PMC9120944; doi:10.3389/fonc.2022.769530)
Supplement: Supplementary file 2 [file Table_2.docx]

**Table S2. Response to first-line therapy in patients with primary ocular adnexal MALT lymphoma**

| **First-line chemotherapy**  **Treatment response** | **Interim response**  **Entire cohort (n=97) Stage IE (n=53)** | | | **Best response during follow-up**  **Entire cohort (n=97) Stage IE (n=53)** | | **Last follow-up**  **Entire cohort (n=97) Stage IE (n=53)** | |
| --- | --- | --- | --- | --- | --- | --- | --- |
| Complete remission | 59 (60.8%) | 32 (60.4%) | | 92 (94.8%) | 49 (92.5%) | 84 (86.6%) | 44 (83.0%) |
| Partial remission | 37 (38.1%) | 20 (37.7%) | | 5 (5.2%) | 4 (7.5%) | 0 (0%) | 0 (0%) |
| Stable disease | 1 (1.0%) | 1 (1.9%) | | 0 (0%) | 0 (0%) | 0 (0%) | 0 (0%) |
| Progressive disease | 0 (0%) | 0 (0%) | | 0 (0%) | 0 (0%) | 0 (0%) | 0 (0%) |
| Relapse* | NA | NA | | 0 (0%) | 0 (0%) | 13 (13.4%) | 9 (17.0%)† |
| **First-line radiotherapy**  **Treatment response** | **Interim response**  **Entire cohort (n=179) Stage IE (n=178)** | | **Best response during follow-up**  **Entire cohort (n=179) Stage IE (n=178)** | | | **Last follow-up**  **Entire cohort (n=179) Stage IE (n=178)** | |
| Complete remission | 177 (98.9%) | 176 (98.9%) | 177 (98.9%) | | 176 (98.9%) | 159 (88.8%) | 158 (88.8%) |
| Partial remission | 1 (0.6%) | 1 (0.6%) | 2 (1.1%) | | 2 (1.1%) | 0 (0%) | 0 (0%) |
| Stable disease | 1 (0.6%) | 1 (0.6%) | 0 (0%) | | 0 (0%) | 0 (0%) | 0 (0%) |
| Progressive disease | 0 (0%) | 0 (0%) | 0 (0%) | | 0 (0%) | 0 (0%) | 0 (0%) |
| Relapse* | NA | NA | | 0 (0%) | 0 (0%) | 20 (11.2%) | 20 (11.2%)‡ |

Sixteen out of 247 limited-stage patients underwent surgical resection of OAML lesions only and achieved CR.

†In chemotherapy group, 2 (15.4%) and 7 (17.5%) patients experienced disease relapse in AJCC/TNM stage T1N0M0 (n=13) and T2~4N0M0 (n=40), respectively.

‡In RT group, 12 (8.8%) and 8 (19.5%) patients experienced disease relapse in AJCC/TNM stage T1N0M0 (n=137) and T2~4N0M0 (n=41), respectively.

*By statistical analysis, the cumulative incidence of relapse between radiotherapy and chemotherapy group showed no significant difference in both entire cohort (16.9% vs. 20.5%, p=0.567) and limited stage (17.1% vs. 21.7%, p=0.485)
