# Supplementary material for: Long-Term Clinical Outcomes in Treatment-Naïve Patients With Orbital Adnexal Mucosa-Associated Lymphoid Tissue Lymphoma: A Single-Center Study
Source: Front Oncol. 2022 May 6;12:769530. doi: 10.3389/fonc.2022.769530 (PMC9120944; doi:10.3389/fonc.2022.769530)
Supplement: Supplementary file 3 [file Table_3.docx]

**Table S3. Adverse events by treatment modalities in entire cohort (n=292)** †

| **First-line radiotherapy (n=179)** | | | **First-line chemotherapy (n=97)** | | | | |
| --- | --- | --- | --- | --- | --- | --- | --- |
| **Adverse events** | **Grade 1-2**  **N (%)** | **Grade 3-4**  **N (%)** | **Adverse events** |  | **Grade 1-2**  **N (%)** | **Grade 3-4**  **N (%)** | |
| Dry eyes | 51 (28.5%) | 19 (10.6%) | **Hematologic** | Neutropenia§ | 24 (24.8%) | 35 (36.1%) | |
| Cataract | 22 (12.3%) | 26 (14.5%) |  | Anemia | 35 (36.1%) | 5 (5.2%) | |
| Cataract diagnosis ≤50yrs | 14 (7.8%) | 9 (5.1%) |  | Thrombocytopenia | 12 (12.4%) | 3 (3.1%) | |
| Cataract surgery | 0 | 20 (11.2%) | **Non-hematologic** | Hepatotoxicity | 33 (34.0%) | 6 (6.2%) | |
| Adnexal inflammation‡ | 28 (15.6%) | 18 (10.1%) |  | Acute renal injury | 5 (5.2%) | 2 (2.1%) | |
| Retinopathy | 12 (6.7%) | 6 (3.4%) |  | Infection | 13 (13.4%) | 0 | |
| Nasolacrimal duct obstruction | 0 | 6 (3.4%) |  | Peripheral neuropathy* | 37 (38.2%) | 3 (3.1%) | |
| **Therapy-related mortality** 0% | | | **Therapy-related mortality** | | | | 0% |

† Sixteen out of 247 limited-stage patients underwent surgical resection of OAML lesions only and achieved CR.

‡ Adnexal inflammation includes keratitis, blepharitis, or conjunctivitis.

§ Among 35 patients experienced Grade III-IV neutropenia during chemotherapy, 9 patients required hospitalization to manage neutropenic fever.
