# Supplementary material for: Long-Term Clinical Outcomes in Treatment-Naïve Patients With Orbital Adnexal Mucosa-Associated Lymphoid Tissue Lymphoma: A Single-Center Study
Source: Front Oncol. 2022 May 6;12:769530. doi: 10.3389/fonc.2022.769530 (PMC9120944; doi:10.3389/fonc.2022.769530)
Supplement: Supplementary file 4 [file Table_4.docx]

**Table S4. Univariate analysis of survival outcomes in primary ocular adnexal MALT lymphoma patients (Entire cohort, n=292)** †

| Variables | OS | *p-*value | PFS | *p-*value | CIR | *p-*value | NRM | *p-*value |
| --- | --- | --- | --- | --- | --- | --- | --- | --- |
| Age (years) |  | 0.053 |  | 0.556 |  | 0.329 |  | *0.017* |
| <60 (n=227) | 98.4% (88.9-99.8) |  | 81.5% (74.0-87.0) |  | 18.5% (12.5-25.3) |  | 0% |  |
| ≥60 (n=65) | 87.5% (38.7-98.1) |  | 75.5% (42.7-91.2) |  | 13.7% (3.2-31.7) |  | 10.8% (0.5-39.8) |  |
| Gender |  | 0.642 |  | 0.857 |  | 0.975 |  | 0.177 |
| Male (n=118) | 95.5% (71.9-99.3) |  | 77.5% (61.7-87.4) |  | 17.9% (9.4-28.7) |  | 4.6% (0.3-19.5) |  |
| Female (n=174) | 98.0% (86.9-99.7) |  | 82.3% (73.3-88.5) |  | 17.6% (10.8-25.6) |  | 0% |  |
| Bilateral lesion |  | 0.417 |  | *0.004* |  | *0.004* |  | 0.679 |
| No (n=203) | 96.3% (86.1-99.1) |  | 86.0% (77.4-91.5) |  | 12.0% (6.9-18.6) |  | 2.0% (0.2-9.2) |  |
| Yes (n=89) | 100% |  | 68.6% (52.1-80.4) |  | 31.0% (17.7-45.4) |  | 0% |  |
| Ann-Arbor stage |  | 0.716 |  | 0.875 |  | 0.904 |  | 0.805 |
| Stage IE (n=247) | 97.1% (88.7-99.3) |  | 80.5% (72.4-86.5) |  | 17.7% (11.9-24.4) |  | 1.7% (0.1-8.1) |  |
| Stage IIE~IV (n=45) | 100% |  | 84.6% (63.6-94.0) |  | 15.4% (4.6-32.1) |  | 0% |  |
| AJCC-TNM stage |  | *0.034* |  | *0.021* |  | *0.037* |  | 0.158 |
| T1~bT1N0M0 (n=159) | 100% |  | 88.0% (80..3-92.8) |  | 11.9% (6.7-18.7) |  | 0% |  |
| Beyond bT1N0M0* (n=133) | 91.1% (68.4-97.7) |  | 68.3% (51.5-80.3) |  | 26.9% (15.8-39.2) |  | 4.9% (0.3-20.7) |  |
| MALT-IPI risk |  | 0.120 |  | 0.127 |  | 0.135 |  | 0.711 |
| Low risk (n=221) | 98.3% (88.4-99.8) |  | 78.5% (69.9-84.9) |  | 19.6% (13.4-26.7) |  | 1.8% (0.1-8.6) |  |
| Intermediate to high risk (n=71) | 90.9% (50.8-98.7) |  | 93.2% (80.2-97.8) |  | 6.8% (1.7-16.9) |  | 0% |  |
| Ki-67 |  | 0.688 |  | 0.434 |  | 0.413 |  | 0.789 |
| <10% (n=229) | 97.0% (88.6-99.2) |  | 81.9% (73.8-87.8) |  | 16.3% (10.7-22.9) |  | 1.7% (0.1-8.3) |  |
| ≥10% (n=63) | 100% |  | 72.7% (43.1-88.6) |  | 27.3% (7.9-51.5) |  | 0% |  |
| Initial treatment modality |  | 0.380 |  | 0.647 |  | 0.567 |  | 0.545 |
| Radiotherapy (n=179) | 96.1% (85.4-99.0) |  | 80.8% (70.8-87.6) |  | 16.9% (10.4-24.7) |  | 2.2% (0.2-10.4) |  |
| Chemotherapy (n=97) | 100% |  | 79.5% (66.2-88.1) |  | 20.5% (10.9-32.1) |  | 0% |  |
| Radiotherapy dose |  | 0.460 |  | 0.481 |  | 0.441 |  | 0.628 |
| <3000cGy (n=86) | 100% |  | 70.2% (36.9-88.2) |  | 29.4% (7.5-56.1) |  | 0% |  |
| ≥3000cGy (n=93) | 95.1% (81.7-98.8) |  | 82.8% (71.3-90.0) |  | 14.5% (7.9-23.0) |  | 2.8% (0.2-12.5) |  |
| Interim response |  | 0.573 |  | 0.648 |  | 0.606 |  | 0.733 |
| CR (n=251) | 96.8% (87.8-99.2) |  | 81.3% (73.0-87.2) |  | 16.9% (11.2-23.6) |  | 1.8% (0.1-8.5) |  |
| SD or PR (n=41) | 100% |  | 77.6% (54.0-90.1) |  | 22.4% (7.7-41.8) |  | 0% |  |

CR, complete remission; CIR, cumulative incidence of relapse; IPI, international prognostic index; NRM, non-relapse mortality; OS, overall survival; PFS, progression-free survival; PR, partial remission; SD, stable disease.

* In the entire cohort, beyond the bT1N0M0 group category also included Ann-Arbor stage II to IV patients.

† All survival outcomes were defined as the time from pathologic diagnosis until each indicated time point (OS; death or the last follow-up, PFS; disease progression, transformation to aggressive lymphoma, relapse, or death, CIR; pathological diagnosis of relapse, NRM; death by any reason without relapse).
