# Supplementary material for: Long-Term Clinical Outcomes in Treatment-Naïve Patients With Orbital Adnexal Mucosa-Associated Lymphoid Tissue Lymphoma: A Single-Center Study
Source: Front Oncol. 2022 May 6;12:769530. doi: 10.3389/fonc.2022.769530 (PMC9120944; doi:10.3389/fonc.2022.769530)
Supplement: Supplementary file 5 [file Table_5.docx]

**Table S5. Univariate analysis of survival outcomes in primary ocular adnexal MALT lymphoma patients (Limited stage IE, n=247)** †

| Variables | OS | *p-*value | PFS | *p-*value | CIR | *p-*value | NRM | *p-*value |
| --- | --- | --- | --- | --- | --- | --- | --- | --- |
| Age (years) |  | *0.011* |  | 0.913 |  | 0.594 |  | *0.007* |
| <60 (n=198) | 98.3% (88.8-99.8) |  | 81.2% (73.4-86.9) |  | 18.7% (12.5-25.8) |  | 0% |  |
| ≥60 (n=53) | 83.3% (27.3-97.5) |  | 69.0% (31.9-88.6) |  | 17.2% (3.9-38.6) |  | 13.8% (0.5-48.1) |  |
| Gender |  | 0.590 |  | 0.777 |  | 0.962 |  | 0.159 |
| Male (n=94) | 94.7% (68.1-99.2) |  | 76.7% (59.3-87.4) |  | 18.2% (9.1-29.8) |  | 5.1% (0.3-21.5) |  |
| Female (n=153) | 98.0% (86.4-99.7) |  | 82.5% (73.0-88.9) |  | 17.4% (10.4-25.9) |  | 0% |  |
| Bilateral lesion |  | 0.415 |  | *0.003* |  | *0.003* |  | 0.696 |
| No (n=177) | 96.1% (85.2-99.0) |  | 86.1% (76.9-91.8) |  | 11.9% (6.5-18.9) |  | 2.0% (0.2-9.6) |  |
| Yes (n=70) | 100% |  | 66.9% (49.1-79.6) |  | 32.7% (18.1-48.1) |  | 0% |  |
| AJCC-TNM stage |  | *0.018* |  | *0.008* |  | *0.018* |  | 0.122 |
| T1~bT1N0M0 (n=159) | 100% |  | 88.0% (80.3-92.8) |  | 11.9% (6.7-18.7) |  | 0% |  |
| Beyond bT1N0M0 (n=88) | 88.9% (61.8-97.2) |  | 64.3% (44.9-78.4) |  | 29.8% (16.6-44.3) |  | 5.8% (0.3-24.2) |  |
| MALT-IPI risk |  | *0.020* |  | 0.277 |  | 0.288 |  | 0.781 |
| Low risk (n=217) | 98.3% (88.4-99.8) |  | 79.4% (70.7-85.7) |  | 18.7% (12.6-25.8) |  | 1.8% (0.1-8.7) |  |
| Intermediate to high risk (n=30) | 83.3% (27.3-97.5) |  | 95.7% (72.9-99.4) |  | 4.3% (0.3-18.7) |  | 0% |  |
| Ki-67 |  | 0.677 |  | 0.193 |  | 0.181 |  | 0.777 |
| <10% (n=194) | 96.8% (87.8-99.2) |  | 82.4% (73.7-88.5) |  | 15.6% (9.8-22.6) |  | 1.9% (0.1-8.8) |  |
| ≥10% (n=53) | 100% |  | 70.1% (41.1-86.8) |  | 29.9% (9.5-53.9) |  | 0% |  |
| Initial treatment modality |  | 0.420 |  | 0.562 |  | 0.485 |  | 0.570 |
| Radiotherapy (n=178) | 96.1% (85.1-99.0) |  | 80.6% (70.4-87.5) |  | 17.1% (10.5-25.0) |  | 2.3% (0.2-10.6) |  |
| Chemotherapy (n=53) | 100% |  | 78.3% (61.9-88.3) |  | 21.7% (10.3-35.7) |  | 0% |  |
| Radiotherapy dose |  | 0.454 |  | 0.500 |  | 0.458 |  | 0.623 |
| <3000cGy (n=86) | 100% |  | 70.2% (36.9-88.2) |  | 29.4% (7.5-56.1) |  | 0% |  |
| ≥3000cGy (n=92) | 95.0% (81.2-98.7) |  | 82.5% (70.8-89.8) |  | 14.7% (8.0-23.3) |  | 2.8% (0.2-12.9) |  |
| Interim response |  | 0.583 |  | 0.857 |  | 0.909 |  | 0.734 |
| CR (n=223) | 96.6% (87.1-99.1) |  | 80.3% (71.5-86.6) |  | 17.7% (11.7-24.8) |  | 1.9% (0.2-9.0) |  |
| SD or PR (n=24) | 100% |  | 82.5% (53.9-94.2) |  | 17.5% (3.8-39.5) |  | 0% |  |

CR, complete remission; CIR, cumulative incidence of relapse; IPI, international prognostic index; NRM, non-relapse mortality; OS, overall survival; PFS, progression-free survival; PR, partial remission; SD, stable disease.

† All survival outcomes were defined as the time from pathologic diagnosis until each indicated time point (OS; death or the last follow-up, PFS; disease progression, transformation to aggressive lymphoma, relapse, or death, CIR; pathological diagnosis of relapse, NRM; death by any reason without relapse).
